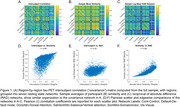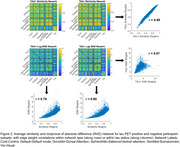# Preliminary Characterization of Participant‐Level Tau Brain Networks in the Health and Aging Brain Study ‐ Health Disparities HABS‐HD Cohort

**DOI:** 10.1002/alz70862_110187

**Published:** 2025-12-23

**Authors:** Evgeny J. Chumin, Alex N Tinnel, Olaf Sporns, Karin L. Meeker, Raul Vintimilla, Sid E. O'Bryant, Andrew J. Saykin

**Affiliations:** ^1^ Indiana University School of Medicine, Indianapolis, IN USA; ^2^ Indiana Alzheimer’s Disease Research Center, Indiana University School of Medicine, Indianapolis, IN USA; ^3^ Center for Neuroimaging, Department of Radiology and Imaging Sciences, Indiana University School of Medicine, Indianapolis, IN USA; ^4^ Indiana University Indianapolis, Indianapolis, IN USA; ^5^ Department of Psychological and Brain Sciences, Indiana University, Bloomington, IN USA; ^6^ Institute for Translational Research, University of North Texas Health Science Center, Fort Worth, TX USA; ^7^ Department of Medical and Molecular Genetics, Indiana University School of Medicine, Indianapolis, IN USA; ^8^ Center for Neuroimaging, Indiana University School of Medicine, Indianapolis, IN USA; ^9^ Department of Radiology and Imaging Sciences, Indiana University School of Medicine, Indianapolis, IN USA

## Abstract

**Background:**

PET neuroimaging is a powerful diagnostic tool that quantifies amyloid and tau accumulation in vivo. Network approaches have been applied to study brain structure and function in Alzheimer’s disease (AD), but it has been challenging to estimate participant‐level networks from PET given the static nature of the data (single value per region), hindering development of multimodal network integration approached in clinical research. Here, we propose a novel framework to derive participant‐level tau similarity networks, from regional PI‐2620 tau PET in 1613 participants from the HABS‐HD study, one of the largest and most diverse community cohorts.

**Method:**

Standardized uptake value ratios (SUVr, normalized to inferior cerebellum) were extracted from 100 cortical regions from the Schaefer functional parcellation. Two types of participant‐level networks were generated: (1) a similarity network, where each connection (edge (i,j)) was computed as 1‐abs(SUVr(i)‐SUVr(j)) and normalized by the maximum difference for each participant, and (2) a reciprocal of absolute difference (RAD) network computed as 1/abs(SUVr(i)‐SUVr(j)). Networks were then averaged across participants and compared against an existing framework of an intersubject correlation (“covariance network”) that is estimated through inter‐regional Pearson correlation of SUVr values across participants. Finally, participants were stratified as Tau+ and Tau‐ based on an SUVr cutoff of 1.1, which was the mean SUVr in Schaefer regions that fall >60% within the tau medial temporal meta‐ROI, with networks generated for each subgroup.

**Result:**

All three network types display a block structure within canonical resting state networks (Figure 1). Edge weight correlations between the covariance network and the two average participant‐level network types were moderate (Pearson r’s 0.49 and 0.32). Average participant‐level networks were highly correlated (r=0.80). Tau positivity stratified networks (Figure 2) were nearly identical for similarity networks, while only moderately correlated for RAD networks (r=0.67).

**Conclusion:**

Normalization in similarity networks would allow for investigations of topological properties of these networks, improving our understanding of AD neurobiology, while RAD networks, which preserve magnitude of SUVr differences, would facilitate patient‐centric multimodal network approaches. These participant‐level PET networks show promise for future integration with MRI brain connectivity networks to develop precision approaches for diagnostic subtyping of AD.